# Psychopathia Sexualis: A Medico-Legal Study

**Published:** 1894-03

**Authors:** 


*Psychopathia Sexualis: A Medico-Legal Study.* By D. R. von KRAFFT-EBING. Authorised Translation of the Seventh Edition, by CHARLES GILBERT CHADDOCK, M.D. Pp. xiv., 436. Philadelphia: The F. A. DAVIS COMPANY. 1893.

This work by the eminent professor in the University of Vienna, better known on the Continent than here, was written to set forth the pathological manifestations of the sexual life, and to attempt to view them from an alienistic point of view, and thereby to find excuses for those strange perversions of that life which from time to time force themselves on public attention, but are well known in the experience of the medical jurist.

It cannot be denied that scientific men have an undoubted right and prerogative to inquire fully into all the strange and curious problems connected with life in its manifestation in the human body, as well as in the mind. Of old time all learned scientific books were written in a dead language "not understanded of the common people"; and, indeed, it is not so long ago that treatises on subjects which it was not desirable should meet the eye of the uninstructed, or of the ordinary reader, were still so veiled. "Democritus Junior," when writing his wonderful work, *The Anatomy of Melancholy*, and treating of "Love Melancholy," was careful to decently clothe his "Subject II." in Latin. In the book before us certain words or small passages are in Latin; but perhaps it would have been better had the greater part of such an extremely unpleasant and repulsive investigation of the morbid deviations of the genital instincts been put into the same language. Especially would this seem to have been needed when we read in the preface that the original work has reached its seventh edition, and it is admitted that such circulation is not a legitimate one, but due to a pornographic interest on the part of the public.

The chapter on "The Psychology of Sexual Life" is an interesting one, and the inquiry into the pathology of the subject opens up some abstruse points. Charcot and Tardieu, as well as many others, have written, and written well, upon these subjects. With regard to this and other chapters the facts must be admitted, but the conclusions drawn must be liable to grave questioning,—as in the case of what the author terms "sexual inversion," and whether it should be treated as a disease or a crime; and where it is urged that some such instances of human depravity should be accepted as being beyond control, and be admitted as necessary conditions of human life, it cannot be conceived that such conclusions could be accepted amongst us. Human passion, when not educated and brought under proper control—whether that control be such as all right-minded men acknowledge as in accordance with the dictates of reason, or whether it be such as has its origin in Divine command,—will run riot, and bring about the mental and moral degradation of the individual; and it seems incredible that if one passion of a base sort is thus allowed to run riot, that Society would be long without allowing others to go that way also. Several cases of pure mental disease, such as are well known and recognised by alienists, are brought forward in this work; but it seems hardly possible that numbers of the other cases given can be classed as other than pure want of moral control due to the negligence of the ordinary rules of chastity and purity.

The book concludes with some short references to the bearing of these cases on jurisprudence.

